# Supplementary material for: Polyketide synthase gene clusters in the ericoid mycorrhizal fungus Oidiodendron maius: genome-wide prediction, expression profiling, and functional characterization of knockout mutants
Source: Front Plant Sci. 2026 Sep 16;17:1924430. doi: 10.3389/fpls.2026.1924430 (PMC13623903; doi:10.3389/fpls.2026.1924430)
Supplement: Supplementary file 1 [file SupplementaryFile1.zip › Supplementary Materials/Supplementary Folder 1.zip!/Oidma1_AssemblyScaffolds_Repeatmasked/knownclusterblast/cluster48/jgi.p_Oidma1_136494_mibig_hits.html]

| MIBiG Protein | Description | MIBiG Cluster | MiBiG Product | % ID | % Coverage | BLAST Score | E-value |
| --- | --- | --- | --- | --- | --- | --- | --- |
| EAU38973.1 | predicted\_protein | BGC0001122 | Isoflavipucine\_biosynthetic\_gene\_cluster | 41 | 96.7880085653 | 337 | 2.4e-92 |
| EAW09119.1 | conserved\_hypothetical\_protein | BGC0000983 | Cytochalasin\_biosynthetic\_gene\_cluster | 40 | 94.4325481799 | 317 | 1.9e-86 |
| EAL85114.1 | alpha/beta\_hydrolase,\_putative | BGC0001037 | Pseurotin\_A\_biosynthetic\_gene\_cluster | 40 | 94.6466809422 | 302 | 6.4e-82 |
| ACS68553.1 | alpha/beta\_hydrolase | BGC0001026 | NG-391\_biosynthetic\_gene\_cluster | 38 | 94.2184154176 | 295 | 1e-79 |
